# Role of stem cells during diabetic liver injury

**DOI:** 10.1111/jcmm.12723

**Published:** 2015-12-09

**Authors:** Ying Wan, Jessica Garner, Nan Wu, Levine Phillip, Yuyan Han, Kelly McDaniel, Tami Annable, Tianhao Zhou, Heather Francis, Shannon Glaser, Qiaobing Huang, Gianfranco Alpini, Fanyin Meng

**Affiliations:** ^1^ ResearchCentral Texas Veterans Health Care SystemTempleTXUSA; ^2^ Department of Internal MedicineScott & White Digestive Disease Research CenterTexas A&M University Health Science Center and Baylor Scott & White HealthcareTempleTXUSA; ^3^ Academic OperationsBaylor Scott & White HealthcareTempleTXUSA; ^4^ Department of PathophysiologyKey Lab for Shock and Microcirculation ResearchSouthern Medical UniversityGuangzhouChina

**Keywords:** stem cells, diabetes, liver diseases, non‐alcoholic fatty liver disease, insulin resistance

## Abstract

Diabetes mellitus is one of the most severe endocrine metabolic disorders in the world that has serious medical consequences with substantial impacts on the quality of life. Type 2 diabetes is one of the main causes of diabetic liver diseases with the most common being non‐alcoholic fatty liver disease. Several factors that may explain the mechanisms related to pathological and functional changes of diabetic liver injury include: insulin resistance, oxidative stress and endoplasmic reticulum stress. The realization that these factors are important in hepatocyte damage and lack of donor livers has led to studies concentrating on the role of stem cells (SCs) in the prevention and treatment of liver injury. Possible avenues that the application of SCs may improve liver injury include but are not limited to: the ability to differentiate into pancreatic β‐cells (insulin producing cells), the contribution for hepatocyte regeneration, regulation of lipogenesis, glucogenesis and anti‐inflammatory actions. Once further studies are performed to explore the underlying protective mechanisms of SCs and the advantages and disadvantages of its application, there will be a greater understand of the mechanism and therapeutic potential. In this review, we summarize the findings regarding the role of SCs in diabetic liver diseases.

## Introduction

The number of patients with diabetes mellitus (DM) is increasing exponentially 1. Diabetes is estimated to be the most common cause of liver diseases 2, 3, which has, in turn, become a major cause of death 4. Of the diabetic population, Type 2 diabetes accounts for more than 90% of the cases. Non‐alcoholic fatty liver disease (NAFLD; including fatty liver, steatohepatitis, liver fibrosis and cirrhosis) 5 is the most common liver disease associated with Type 2 diabetes. In addition, Type 1 diabetes is also linked to increased risk of chronic liver injury 6, 7. Epidemiological studies demonstrate that diabetic patients are at higher risk of developing chronic liver disease and hepatocellular carcinoma (HCC) 8, 9, 10, 11, which has major implications on long‐term health. With these statistics in mind, research aimed at developing therapeutic interventions is of great interest.

There is rising evidence that stem cells (SCs), such as mesenchymal stem cells (MSCs) derived from bone marrow, could serve as alternative therapies for diabetic liver damage. In fact, studies suggest that SCs may be a novel and promising therapeutic approach of cell‐based therapy for diabetic liver injury. To understand the potential curative role of SCs in diabetic liver injury, we highlighted the latest development in the mechanisms of hepatocyte damage during diabetes and the application of SCs in diabetic liver diseases.

## Pathological features of diabetic liver injury

When diabetic liver injury is present, several pathological changes are observed in the liver: (*i*) Excessive glycogen deposits in hepatocytes, including the cytoplasm and nucleus, and results in a visible nuclear glycogen cavity. Hepatic glycogenosis is a hepatic complication of diabetes that primarily occurs in patients with Type 1 diabetes 12. (*ii*) Steatosis is present. Hepatocytes ballooning is observed and Kupffer cells are filled with lipid droplets 13. Steatosis or abnormal retention of lipids in the liver may be focal or diffuse. Hepatocyte steatosis is an important feature of diabetic liver diseases, which sometimes leads to formation of a fatty granuloma. (*iii*) The formation of Mallory–Denk bodies is observed. Mallory–Denk bodies are round or irregular hyaline material in hepatocytes plasma. These are typical characteristic changes observed in diabetic liver diseases. (*iv*) Inflammatory cells infiltration is evident. Mixed inflammatory cells infiltrate the lobule and portal area, including mononuclear cells and a few neutrophils. (*v*) Interstitial fibrotic proliferation, mainly around the central vein, is obvious which is considered as another important characteristic change in diabetic liver injury 14. Liver fibrosis in NAFLD secondary to Type 2 diabetes features the deposition of collagen fibres, initially occurring in the central vein and around sinus gap. With disease progression, portal fibrosis develops and eventually leads to cirrhosis. (*vi*) Microangiopathy may occur. For example, abnormal capillary thickness occurs from deposition of periodic acid‐Schiff stain positive materials in endothelial cells 15, resulting in endothelial cell proliferation, swelling, damage and vascular leakage. Other pathologic features such as intrahepatic cholestasis and fat necrosis may be present in the liver of the patients with diabetes. Fat necrosis can lead to cirrhosis due to severe diabetic liver injury 16 and is associated with a poor prognosis.

## Mechanisms involved in the development of diabetic liver injury

### Insulin resistance with diabetic liver injury

Insulin resistance (IR) could have a major role in underlying liver diseases secondary to Type 2 diabetes. The relationship between IR and fatty liver disease has been recognized in patients with Type 2 diabetes. The liver is a target organ for insulin and has a vital role in regulating lipid metabolism by keeping the balance between circulating and stored lipids. Insulin is well known to promote the production of fatty acids in the liver. This is crucial since fatty acids are an important source of fuel for metabolism and fatty acids are released from the liver as lipoproteins (in varying densities), which provide free fatty acids (FFA) for other tissues. Hormone‐sensitive triglyceride lipase (HSL) is an important enzyme in mobilizing stored fat. Hormone‐sensitive triglyceride lipase is up‐regulated by catecholamines or adrenocorticotropic hormone and is down‐regulated by insulin. Hence, during periods of stress when fatty acids for fuel are needed, insulin is suppressed and HSL activity is high. This leads to the release of FFA, which can be utilized for cellular energy through Adenosine Triphosphate (ATP) production. In IR, HSL activity is uninhibited resulting in increased fat mobilization. This leads to increased levels of FFA circulating in the serum and FFA entering the liver. Upon entering the liver, these FFAs lead to the formation of a large amount triglyceride deposition in the liver. This triglyceride deposition contributes to hepatocyte degeneration and fatty liver disease.

In liver diseases, IR has been recognized as an independent predictor and risk factor for the development of alcoholic and non‐alcoholic steatohepatitis, chronic viral hepatitis and HCC 17, 18, 19.

### Oxidative stress with diabetic liver injury

During the early onset of diabetes, oxidative stress occurs and progressively increases. Increased oxidative stress is believed to play a crucial role in the aetiology and pathogenesis of chronic complications of diabetes in diverse tissues 20, 21, 22. The link has been described between oxidative stress and the pathological alterations in liver morphology and function that are observed in diabetes 23. Streptozotocin (STZ), a glucosamine‐nitrosourea compound, is a naturally occurring compound that increases lipid peroxidation resulting in peroxide‐mediated damage of beta cells in the pancreas of mammals. Streptozotocin is used for the induction of diabetes in rats by lipid peroxide‐mediated damage.

The liver is the main organ for detoxifying and oxidative processes, and at the early stage of experimental STZ‐induced diabetes, biomarkers of oxidative stress are increased in the liver 24. Oxidative stress‐mediated damage to liver tissue has been identified leading to non‐alcoholic steatohepatitis in diabetes and HCC 4. The importance of liver oxidative stress in diabetic liver injury is further confirmed by experimental and clinical data 25, 26, 27, 28. Oxidative stress is an imbalance between the generation of reactive oxygen species (ROS) and the cell's ability to detoxify these reactive intermediates resulting in cellular damage. Generated oxidative stress *via* different mechanisms in the liver leads to negative effects 29. In diabetes, increased ROS come from multiple sources including enzymatic, non‐enzymatic and mitochondrial respiratory chain reactions 30. On the other hand, there are suppressed activities of the antioxidants in diabetes. The principal antioxidant enzymes and non‐enzymatic antioxidants that play an important role in scavenging ROS and preventing oxidative stress include: superoxide dismutases (SOD), catalases (CAT), small molecules such as reduced glutathione, vitamins C and E and other compounds. Unfortunately, diabetic patients with IR are susceptible to hyperglycaemia and hyperlipidemia. This causes decreased activity of antioxidant enzymes SOD, CAT, glutathione peroxidase *in vivo* and decreased radical scavenging ability, resulting in oxidative stress. Enhanced oxidative stress induces lipid peroxidation and damage of cell membrane and organelle membrane such as mitochondrial membrane. The damage of mitochondrial membrane further exacerbates respiratory chain dysfunction and decreased synthesis of H^+^‐ATP enzyme 31. All of this leads to hepatocytes dysfunction in the process of FFA oxidation resulting in excessive triglyceride deposition in the liver or fatty liver.

As oxidative stress has been shown to be important in the pathogenesis of diabetic liver diseases 32, strategies to decrease oxidative stress have been considered for diabetes management 33. And these strategies were identified to be effective in protection of liver function in diabetes 34, 35.

### Endoplasmic reticulum stress and diabetic liver injury

The endoplasmic reticulum (ER) is the main place where protein synthesis, or protein folding, takes place in cells and is extremely sensitive to many stimuli. The liver is a major target organ for glucose and lipid metabolism; thus, there is an abundant amount of ER. Several factors, including glucose and lipid disorders, can disrupt the cells ER homoeostasis by resulting in misfolded proteins. When cells are exposed to glucose or lipid metabolism disorders, free radicals, oxidative stress and other factors ER stress (ERS) or unfolded protein response is induced 36. This response is an adaptive mechanism that is meant to maintain the homoeostasis within the cells ER *via* several mechanisms, including degradation of misfolded proteins. When this mechanism fails to restore homoeostasis within the cell, it induces apoptosis. In fact, chronic activation of the ERS has been liked to inflammation, steatosis and cell injury. Studies demonstrated that ERS is involved in the pathogenesis of several liver diseases including NAFLD, liver cirrhosis and viral hepatitis 37, 38. This response plays an important role to exacerbate lipid metabolic disorder and contributes to steatohepatitis in diabetes 39.

### Other mechanisms related to diabetic liver injury

Hyperlipidemia and hyperglycaemia in diabetes induces transcription of proinflammatory cytokines, tumour necrosis factor alpha (TNF‐α) and monocyte chemotactic protein‐1 (MCP‐1). They also induce transcription of the adipokine, fatty acid‐binding protein 4 (FABP4), by nuclear translocation of NF‐κB (nuclear factor kappa light chain enhancer of activated B cells). Transcription of this adipokine and pro‐inflammatory cytokines results in hepatic injury and IR 40, 41. In diabetes, there is also excessive infiltration of bone marrow‐derived cells (BMDCs) into the liver. This infiltrative process causes parenchymal cells to produce pro‐insulin and cytotoxic TNF‐α, which leads to degeneration or apoptosis of hepatocytes.

In addition, microcirculation dysfunction from the thickening of the blood capillary basement membrane causes disruption of oxygen diffusion resulting in hypoxia. As a result, hypoxia leads to degeneration and necrosis of hepatocytes resulting in liver dysfunction. Other factors or mechanisms that play a role in the development of diabetic liver diseases seen in Type 2 diabetes include: leptin, adiponectin and resistin.

## Categorizations of stem cells

Stem cells refer to cells that possess high potencies of self‐renewal and differentiation, meaning that they can differentiate into many specific cell types 42, 43. Proliferation and directed differentiation of SCs has extensive application value 44, 45, 46. It has been reported that SCs may have protective effects in the process of liver injury and hepatocyte regeneration. Stem cells can differentiate into liver SCs and liver cells under certain circumstances resulting in repair of liver injury and liver reconstruction.

Based on their origin and functional properties, SCs are classified into three categories: embryonic SCs (ESCs), induced pluripotent SCs (iPSCs) and adult SCs. Adult SCs can also be subdivided into the following categories: hematopoietic SCs, MSCs and endothelial progenitor cells (EPCs) 47. Embryonic stem cells have the potential of differentiating into numerous types of cells in the body. These include all three germ layers cells both *in vivo* and *in vitro* 48, 49. These cells are pathogen‐free and can be engineered for different purposes as long as the right environment and precursors are provided for their growth 49. Compared to ESCs, adult SCs have a lower capability of proliferation, differentiation and regeneration of tissues 50. In research, the benefits of using adult SCs are void of the ethical problems that are often associated with human ESCs (hESCs). β‐cell neogenesis in adults has been shown in animal models of experimentally induced pancreatic damage, suggesting the presence of adult SCs or progenitor cells 51. Of particular interest, hepatic oval cells (HOCs) are facultative progenitor cells in the liver that have the potential to trans‐differentiate into hepatocytes, cholangiocytes and pancreatic endocrine cells 52, 53 in the setting of severe liver injury. Research has demonstrated that relying on histological localization to identify HOCs runs the risk of including hematopoietic and other cells in the analysis due to similar surface markers 100. Several markers have been identified various research studies that help select HOCs during laboratory isolation including: EpCam, E‐cadherin, CD133^+^ and CD29^+^. Meanwhile, the interplay of the ductular reaction and fibrosis in NAFLD has been investigated. A close association between the expansion of HOCs and the ductular reaction in liver biopsy specimens of NASH has been verified. The degree of ductular reactions is significantly correlated with the degree of fibrosis, which involves direct epithelial mesenchymal transition of the cholangiocytes to myofibroblasts 54. Last, iPSCs are found to be generated from fibroblast cells by fibroblast specific transcription factor transduced into them 55.

## Stem cells and diabetes

In recent years, transplantation of SCs has been demonstrated as an effective treatment for diabetes 56. Embryonic stem cells have the highest differentiation potential into insulin‐producing cells 57. Soria *et al*. published the first report in 2000 about insulin‐producing cells from mouse ESCs, but these cells had a short life span 58. Since then, more studies have been performed in this field. Many researchers found that MSCs can also differentiate into insulin‐producing cells in special conditions 59, 60. Other studies demonstrated that several cytokines with immunomodulation and nutrition ability were secreted, which can contribute to the activity and function of islet beta cells when MSCs were co‐cultured with islet beta cells *in vitro* or co‐transplantation of them *in vivo* 61, 62. Several laboratory and clinical studies have shown that MSCs have immunomodulation ability by regulating the activity of B cells, T cells, Natural Killer cells and cytokines, transforming growth factor beta and interleukin (IL)‐10 63. These cells could potentially differentiate into insulin‐producing cells in special cultures. The types of MSCs that demonstrate the ability to differentiate into insulin‐producing cells *in vitro* include: MSCs derived from bone marrow 64, 65, adipose tissue 66 and umbilical cord cells 67. Usage of both bone marrow MSCs and umbilical cord MSCs had positive effects in animal studies with evidence of improved blood glucose status 68, 69, 70, 71. Human iPSCs can be differentiated into insulin‐producing cells upon step‐wise differentiation protocols into SOX17‐positive cells, PDX1‐positive cells (pancreatic progenitors) and NGN3‐positive cells (endocrine progenitors) 72, 73, 74. In addition, other different types of cells including skin fibroblast cells 75, human neural progenitor cells 76, HOCs 52 and placenta‐ derived SCs 77 have the potential to differentiate into insulin‐producing cells in special conditions. Of note, several groups have made the tremendous progress in improving strategies to generate potentially functional insulin‐producing cells 78. Stem cell‐based therapies have emerged as a potent strategy for treatment of diabetes and diabetic complications such as diabetic foot 79, 80, 81, 82.

## Stem cells in diabetic liver injury

Liver transplant as a single therapeutic modality for improved survival in end stage liver disease patients is becoming more problematic due to a shortage of liver donors, high cost, chronic immunosuppression and associated complications. As a result, a number of studies are being conducted using cell base therapy as an alternative. More and more studies have been published showing that cell‐based therapy, especially therapy using bone marrow cells, is an alternative to improve liver function in the setting of liver injury 83, 84, 85, 86. One study looked at several types of bone marrow cells (hematopoietic SCs, MSCs and mononuclear cells) to determine which type of cell had the biggest contribution to liver regeneration in hepatic injury based on Green Fluorescent Protein (GFP) fluorescence in injured liver tissue 81. Their results showed the MSC transplanted group had the highest GFP fluorescence suggesting MSCs have the highest potential for regeneration of injured liver tissue 81. Another study found that human umbilical cord matrix stem cell (hUCMSC) transplantation regulates systemic cytokine profiles and may reverse hepatic injury. In hepatocyte injury, hUCMSCs suppress the expression of proinflammatory cytokine IL‐6 resulting in enhancement of recovery and proliferation of injured hepatocytes *in vitro*. In CCl_4_‐injured mice, hUCMSCs ameloriated mouse hepatic injury *in vivo* by decreasing inflammation, apoptosis and denaturation, an increasing proliferation and recovery of hepatocytes 82. An issue in using MSCs in repair of liver damage is their homing and survival in transplant recipient organs. A study demonstrated that synergistic treatment of fibrotic liver with MSCs and nitric oxide enhances homing of transplanted cells resulting in significant decreases in fibrosis 83. The prosed mechanism of nitric oxide in this study is that nitric oxide is an apoptotic inducer of activated hepatic stellate cells, which have a well known role in inducing fibrosis in response to liver injury 83. Stimulation of endogenous regeneration represents another potential mechanism of an MSC‐based therapeutic effect, previously described in models of myocardial infarction.

To date, some studies suggest there is potential value of SCs in diabetic liver damage. Liver dysfunction is increased in the setting of hyperglycaemia. Previous studies demonstrated improvement of blood glucose levels following pancreatic β‐cell regeneration 87, 88 by using MSCs suggesting that amelioration of hyperglycaemia improves liver dysfunction. Other researchers have shown that MSC therapy reversed hepatic injury in high fat diet (HFD)‐induced diabetic mice by suppressing pro‐inflammatory cytokine expression in the liver. However, MSC therapy failed to reverse obesity, hypercholesterolaemia and hyperglycaemia 89, 90. Mesenchymal stem cells derived from different tissues can generate hepatocyte‐like cells *in vitro*, but are not fully functional, mature or transplantable equivalents of hepatocytes isolated from an adult liver 91. The mechanism underlying MSC therapy is suggested to mediate the cell complement effect by cell differentiation or various paracrine effects through humoural factors secreted by MSCs 92.

Recently, researchers have shown that the curative effects of MSCs conditional medium (MSC‐CM) and MSC therapies were similar on damaged hepatocytes in diabetic mice. In diabetes, hepatic injury is mediated by the accelerated infiltration of BMDCs into the liver producing cytotoxic chemokines leading to IR, apoptosis and fatty degeneration of hepatocytes. Mesenchymal stem cells and MSC‐CM protect against hepatic injury by decreasing the abnormal infiltration of BMDCs into the liver by suppressing the release of markers that mediate this infiltration (*i.e*. IL‐6, CD11c, Fizz1, ICAM‐1) and cytotoxic chemokine expression (*i.e*. TNF‐α, MCP‐1, TLR‐4) through humoural factors secreted by MSCs 91. The study showed that although hyperglycaemia was not corrected, diabetes‐induced liver dysfunction was markedly reversed by MSCs and MSC‐CM therapies. Both therapies are powerful tools for repairing liver injury 53. Hepatocyte regeneration was observed immediately following intravenous administration of MSCs or MSC‐CM in STZ‐diabetic mice despite hyperglycaemic conditions 53. Hepatocyte regeneration is due to a wide variety of growth factors and cytokines synthesized by MSCs that have paracrine effects on local cellular dynamics. Mesenchymal stem cell and MSC‐CM therapies ameliorated IR and the homoeostasis model assessment‐estimated IR was down‐regulated in HFD‐diabetic mice 53.

Another mechanism of hepatocyte repair observed in MSC and MSC‐CM therapy includes reduction in lipid accumulation and inhibiting expression of pro‐inflammatory cytokines in diabetic liver injury. The inhibition of pro‐inflammatory cytokines leads to decreased activation of c‐Jun amino‐terminal kinase and p38 mitogen‐activated protein kinase, which reduces hepatocyte apoptosis. Mesenchymal stem cells and MSC‐CM also prevent liver fibrosis 53. These mechanisms of repair demonstrate that MSC therapy may be a novel remedy in diabetic hepatocyte injury.

As previously mentioned, HOCs are thought to be the primary multipotent SCs population in the liver. Hepatic oval cell activation and trans‐differentiation into pancreatic β‐cells reversed hyperglycaemia and ameliorated the diabetic liver injury in one study 52. In another study, HOCs differentiated into insulin‐producing pancreatic islet cells following culture in high glucose media 101. Hepatic oval cells may be promising in the therapy of diabetic liver injury. Also, a recent study verified that SC transplantation up‐regulated haem oxygenase‐1 (HO‐1) and 5′‐AMP activated protein kinase, and increased the expression of Sirt1, which ameliorated fatty liver in Type 2 diabetic mice 93.

In summary, the mechanisms underlying the curative effect of SCs on diabetic liver injury may include the following: SCs reduce hyperglycaemia and alleviate liver damage; they directly differentiate into islet beta cells or hepatocytes under certain conditions; they induce endogenous SCs to regenerate, such as promoting HOCs to differentiate into fully functional mature hepatic cells; they adjust the immune response, lipogenesis and relieve IR and they may exert an anti‐apoptotic effect and prevent liver fibrosis.

## Limitations and issues of stem cell treatment

Although SC therapy has many advantages, adverse reactions and risk do exist. High immune reactions and ethical concerns have limited the application of ESCs. Some studies have reported that MSCs have different effects on tumour cells *in vivo* and *in vitro* 94, 95, 96. *In vivo*, MSCs contribute to tumorigenesis and tumour metastasis 94, 95. The aetiology of this phenomenon may be related to MSCs' ability of differentiation when the host immunity is relatively low 97 and tumour angiogenesis promoted by MSCs. However, MSCs could inhibit the proliferation and invasion of tumour cells while increasing the apoptosis of tumour cells *in vitro* 98. The promotion of tumorigenicity and metastasis has limited the clinical application of MSCs. The use of SCs is also limited by their potential to induce differentiation of cells into to bone, cartilage, adipose tissue, *etc*. 99. They have the ability to impact organ function and other systems, including the immune system 100. Therefore, further studies are needed to effectively demonstrate that the probability of tumorigenesis can be reduced or avoided. Prior to clinical application, MSCs will be strict screened and monitored to ensure its safety and effectiveness.

## Conclusion

**Figure 1:**
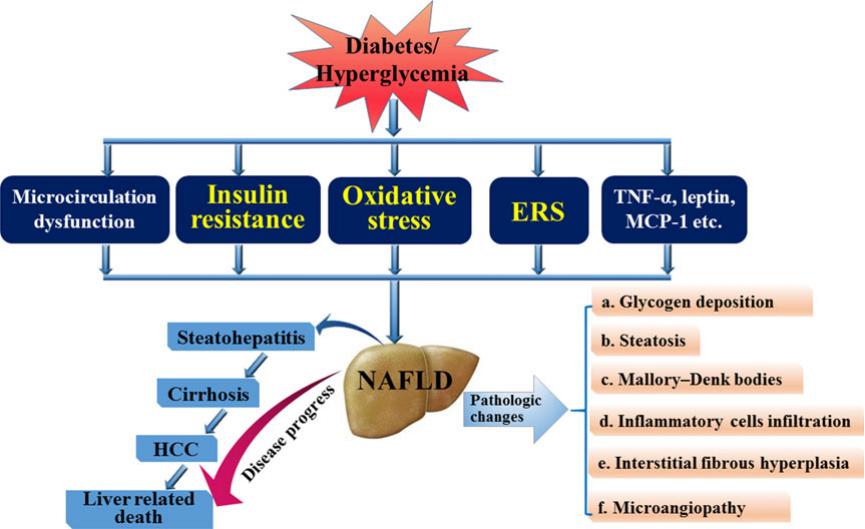
The mechanisms and pathological changes of liver injury induced by diabetes. Diabetes may result in diabetic liver injury, in which the most common is non‐alcoholic fatty liver disease (NAFLD) that can develop into steatohepatitis, cirrhosis, hepatocellular carcinoma (HCC) and can even lead to death. When NAFLD is present, there are many pathological changes in the liver including glycogen deposition in hepatocytes, hepatocyte fatty degeneration, formation of Mallory–Denk bodies, interstitial fibrous hyperplasia, *etc*. The mechanisms involved in the development of diabetic liver injury include insulin resistance (IR), oxidative stress, endoplasmic reticulum stress (ERS), inflammatory cytokines and so on.

**Figure 2:**
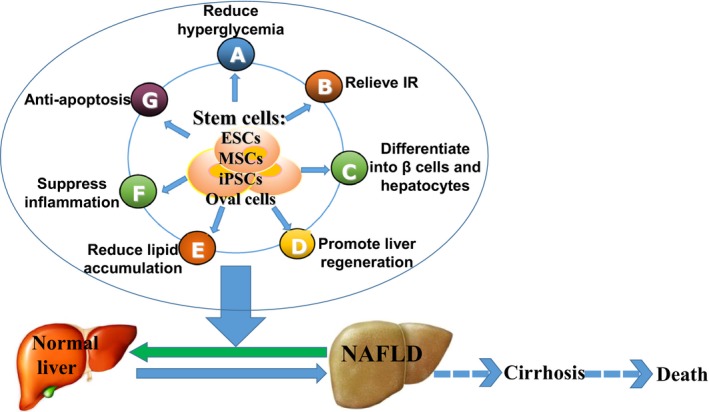
The mechanisms related to the role of stem cells in diabetic liver injury. Stem cell treatment for diabetic liver injury contributes to the improvement of liver function and histology, for example, embryonic stem cells (ESCs), mesenchymal stem cells (MSCs), induced pluripotent stem cells (iPScs) and hepatic oval cells. The underlying mechanisms of this action may include reducing hyperglycaemia, differentiating into β‐cells and hepatocytes, relieving insulin resistance (IR), reducing lipid accumulation and anti‐apoptosis, *etc*.

The prevalence of diabetes and the lack of donor livers for transplantation have prompted experimental research in the field of SC therapy for diabetes and diabetic liver injury. Diabetes, especially Type 2 DM, leads to diabetic liver diseases, mostly common for NAFLD, by the mechanisms involving IR, oxidative stress, endoplastic reticulum stress, cytokines such as TNF‐α, *etc*. (Fig. 1). Stem cells are cells with multi‐directional differentiation potential. And the advance in the research of SCs has fueled increasing hopes of SCs as an effective therapy alteration for diabetic liver injury on the basis of reducing hyperglycemia, differentiating into β‐cells and hepatocytes, relieving IR, reducing lipid accumulation, promoting endogenous liver regeneration *etc*. (Fig. 2). Until now, most research has been focused on MSCs and their impact on diabetic liver. Mesenchymal stem cells are an invaluable source of SCs that are easily accessible from a variety of postnatal tissues. Mesenchymal stem cells have been identified as effective in Phases I to IV of clinical trials for the treatment of Type 1 and 2 DM. In addition, iPSCs have been shown to have an embryonic‐like pluripotent state that may differentiate into any type of human cells. Diabetic hepatocyte damage may be improved by SC therapy, which is promising; however, further efforts are needed to explore the safety, efficacy and avoidance of tumour‐genesis. If proven to be safe, SC therapy for diabetic liver damage may offer a novel therapy in the management of patients with diabetic liver diseases.

## Conflicts of interest

All authors declare that they have no conflicts of interests.

## References

[1] James PT , Rigby N , Leach R . The obesity epidemic, metabolic syndrome and future prevention strategies. Eur J Cardiovasc Prev Rehabil. 2004; 11: 3–8. 15167200 10.1097/01.hjr.0000114707.27531.48

[2] Guven A , Yavuz O , Cam M , *et al* Effects of melatonin on streptozotocin‐induced diabetic liver injury in rats. Acta Histochem. 2006; 108: 85–93. 16714049 10.1016/j.acthis.2006.03.005

[3] Baig NA , Herrine SK , Rubin R . Liver disease and diabetes mellitus. Clin Lab Med. 2001; 21: 193–207. 11321935

[4] Tolman KG , Fonseca V , Dalpiaz A , *et al* Spectrum of liver disease in type 2 diabetes and management of patients with diabetes and liver disease. Diabetes Care. 2007; 30: 734–43. 17327353 10.2337/dc06-1539

[5] Holstein A , Hinze S , Thiessen E , *et al* Clinical implications of hepatogenous diabetes in liver cirrhosis. J Gastroenterol Hepatol. 2002; 17: 677–81. 12100613 10.1046/j.1440-1746.2002.02755.x

[6] Olsson R , Wesslau C , William‐Olsson T , *et al* Elevated aminotransferases and alkaline phosphatases in unstable diabetes mellitus without ketoacidosis or hypoglycemia. J Clin Gastroenterol. 1989; 11: 541–5. 2507626 10.1097/00004836-198910000-00010

[7] Torbenson M , Chen YY , Brunt E , *et al* Glycogenic hepatopathy: an underrecognized hepatic complication of diabetes mellitus. Am J Surg Pathol. 2006; 30: 508–13. 16625098 10.1097/00000478-200604000-00012

[8] El‐Serag HB , Tran T , Everhart JE . Diabetes increases the risk of chronic liver disease and hepatocellular carcinoma. Gastroenterology. 2004; 126: 460–8. 14762783 10.1053/j.gastro.2003.10.065

[9] Fracanzani AL , Valenti L , Bugianesi E , *et al* Risk of severe liver disease in nonalcoholic fatty liver disease with normal aminotransferase levels: a role for insulin resistance and diabetes. Hepatology. 2008; 48: 792–8. 18752331 10.1002/hep.22429

[10] Davila JA , Morgan RO , Shaib Y , *et al* Diabetes increases the risk of hepatocellular carcinoma in the United States: a population based case control study. Gut. 2005; 54: 533–9. 15753540 10.1136/gut.2004.052167 PMC1774454

[11] Veldt BJ , Chen W , Heathcote EJ , *et al* Increased risk of hepatocellular carcinoma among patients with hepatitis C cirrhosis and diabetes mellitus. Hepatology. 2008; 47: 1856–62. 18506898 10.1002/hep.22251

[12] Julian MT , Alonso N , Ojanguren I , *et al* Hepatic glycogenosis: an underdiagnosed complication of diabetes mellitus? World J Diabetes. 2015; 6: 321–5. 25789113 10.4239/wjd.v6.i2.321 PMC4360425

[13] Yamaguchi K , Yang L , McCall S , *et al* Inhibiting triglyceride synthesis improves hepatic steatosis but exacerbates liver damage and fibrosis in obese mice with nonalcoholic steatohepatitis. Hepatology. 2007; 45: 1366–74. 17476695 10.1002/hep.21655

[14] Lefkowitch JH . Hepatobiliary pathology. Curr Opin Gastroenterol. 2007; 23: 221–31. 17414836 10.1097/MOG.0b013e3280adc92e

[15] Teoh SL , Latiff AA , Das S . A histological study of the structural changes in the liver of streptozotocin‐induced diabetic rats treated with or without Momordica charantia (bitter gourd). Clin Ter. 2009; 160: 283–6. 19795081

[16] Marchesini G , Brizi M , Bianchi G , *et al* Nonalcoholic fatty liver disease: a feature of the metabolic syndrome. Diabetes. 2001; 50: 1844–50. 11473047 10.2337/diabetes.50.8.1844

[17] Takamatsu S , Noguchi N , Kudoh A , *et al* Influence of risk factors for metabolic syndrome and non‐alcoholic fatty liver disease on the progression and prognosis of hepatocellular carcinoma. Hepatogastroenterology. 2008; 55: 609–14. 18613418

[18] Harrison SA . Insulin resistance among patients with chronic hepatitis C: etiology and impact on treatment. Clin Gastroenterol Hepatol. 2008; 6: 864–76. 18585970 10.1016/j.cgh.2008.03.024

[19] Marchesini G , Marzocchi R . Metabolic syndrome and NASH. Clin Liver Dis. 2007; 11: 105–17, ix. 17544974 10.1016/j.cld.2007.02.013

[20] Raza H , Prabu SK , John A , *et al* Impaired mitochondrial respiratory functions and oxidative stress in streptozotocin‐induced diabetic rats. Int J Mol Sci. 2011; 12: 3133–47. 21686174 10.3390/ijms12053133 PMC3116180

[21] Yanardag R , Ozsoy‐Sacan O , Orak H , *et al* Protective effects of glurenorm (gliquidone) treatment on the liver injury of experimental diabetes. Drug Chem Toxicol. 2005; 28: 483–97. 16298877 10.1080/01480540500262961

[22] Maritim AC , Sanders RA , Watkins JB , *et al* Diabetes, oxidative stress, and antioxidants: a review. J Biochem Mol Toxicol. 2003; 17: 24–38. 12616644 10.1002/jbt.10058

[23] Grigorov I , Bogojevic D , Jovanovic S , *et al* Hepatoprotective effects of melatonin against pronecrotic cellular events in streptozotocin‐induced diabetic rats. J Physiol Biochem. 2014; 70: 441–50. 24604251 10.1007/s13105-014-0322-7

[24] Kakkar R , Mantha SV , Radhi J , *et al* Increased oxidative stress in rat liver and pancreas during progression of streptozotocin‐induced diabetes. Clin Sci (Lond). 1998; 94: 623–32. 9854460 10.1042/cs0940623

[25] Rutter GA . Diabetes: the importance of the liver. Curr Biol. 2000; 10: R736–8. 11069096 10.1016/s0960-9822(00)00737-5

[26] Evelson P , Susemihl C , Villarreal I , *et al* Hepatic morphological changes and oxidative stress in chronic streptozotocin‐diabetic rats. Ann Hepatol. 2005; 4: 115–20. 16010244

[27] Galley HF . Oxidative stress and mitochondrial dysfunction in sepsis. Br J Anaesth. 2011; 107: 57–64. 21596843 10.1093/bja/aer093

[28] Unal D , Aksak S , Halici Z , *et al* Effects of diabetes mellitus on the rat liver during the postmenopausal period. J Mol Histol. 2011; 42: 273–87. 21604006 10.1007/s10735-011-9331-9

[29] Cavadas LF , Nunes A , Pinheiro M , *et al* [Management of menopause in primary health care]. Acta Med Port. 2010; 23: 227–36. 20470470

[30] Haidara MA , Yassin HZ , Zakula Z , *et al* Diabetes and antioxidants: myth or reality? Curr Vasc Pharmacol. 2010; 8: 661–72. 19485907 10.2174/157016110792006941

[31] Waisundara VY , Hsu A , Tan BK , *et al* Baicalin reduces mitochondrial damage in streptozotocin‐induced diabetic Wistar rats. Diabetes Metab Res Rev. 2009; 25: 671–7. 19688721 10.1002/dmrr.1005

[32] Matsunami T , Sato Y , Ariga S , *et al* Regulation of oxidative stress and inflammation by hepatic adiponectin receptor 2 in an animal model of nonalcoholic steatohepatitis. Int J Clin Exp Pathol. 2010; 3: 472–81. 20606728 PMC2897102

[33] Nozik‐Grayck E , Suliman HB , Piantadosi CA . Extracellular superoxide dismutase. Int J Biochem Cell Biol. 2005; 37: 2466–71. 16087389 10.1016/j.biocel.2005.06.012

[34] Ortiz‐Avila O , Gallegos‐Corona MA , Sanchez‐Briones LA , *et al* Protective effects of dietary avocado oil on impaired electron transport chain function and exacerbated oxidative stress in liver mitochondria from diabetic rats. J Bioenerg Biomembr. 2015; 47: 337–53. 26060181 10.1007/s10863-015-9614-z

[35] Sila A , Kamoun Z , Ghlissi Z , *et al* Ability of natural astaxanthin from shrimp by‐products to attenuate liver oxidative stress in diabetic rats. Pharmacol Rep. 2015; 67: 310–6. 25712656 10.1016/j.pharep.2014.09.012

[36] Cunard R , Sharma K . The endoplasmic reticulum stress response and diabetic kidney disease. Am J Physiol Renal Physiol. 2011; 300: F1054–61. 21345978 10.1152/ajprenal.00021.2011 PMC3094049

[37] Canbay A , Friedman S , Gores GJ . Apoptosis: the nexus of liver injury and fibrosis. Hepatology. 2004; 39: 273–8. 14767974 10.1002/hep.20051

[38] Shiraishi H , Okamoto H , Yoshimura A , *et al* ER stress‐induced apoptosis and caspase‐12 activation occurs downstream of mitochondrial apoptosis involving Apaf‐1. J Cell Sci. 2006; 119: 3958–66. 16954146 10.1242/jcs.03160

[39] Zhang Q , Li Y , Liang T , *et al* ER stress and autophagy dysfunction contribute to fatty liver in diabetic mice. Int J Biol Sci. 2015; 11: 559–68. 25892963 10.7150/ijbs.10690 PMC4400387

[40] Tilg H . The role of cytokines in non‐alcoholic fatty liver disease. Dig Dis. 2010; 28: 179–85. 20460908 10.1159/000282083

[41] Solomon SS , Odunusi O , Carrigan D , *et al* TNF‐alpha inhibits insulin action in liver and adipose tissue: a model of metabolic syndrome. Horm Metab Res. 2010; 42: 115–21. 19960405 10.1055/s-0029-1241834

[42] Yao P , Zhan Y , Xu W , *et al* Hepatocyte growth factor‐induced proliferation of hepatic stem‐like cells depends on activation of NF‐kappaB. J Hepatol. 2004; 40: 391–8. 15123351 10.1016/j.jhep.2003.11.001

[43] Hu AB , He XS , Cai JY , *et al* Hepatic differentiation of mouse ES cells into BE cells *in vitro* . Cell Biol Int. 2006; 30: 459–65. 16600644 10.1016/j.cellbi.2006.01.006

[44] Cantz T , Sharma AD , Jochheim‐Richter A , *et al* Reevaluation of bone marrow‐derived cells as a source for hepatocyte regeneration. Cell Transplant. 2004; 13: 659–66. 15648736 10.3727/000000004783983521

[45] Snykers S , De Kock J , Vanhaecke T , *et al* Differentiation of neonatal rat epithelial cells from biliary origin into immature hepatic cells by sequential exposure to hepatogenic cytokines and growth factors reflecting liver development. Toxicol In Vitro. 2007; 21: 1325–31. 17507196 10.1016/j.tiv.2007.03.013

[46] Wollert KC , Meyer GP , Lotz J , *et al* Intracoronary autologous bone‐marrow cell transfer after myocardial infarction: the BOOST randomised controlled clinical trial. Lancet. 2004; 364: 141–8. 15246726 10.1016/S0140-6736(04)16626-9

[47] Godfrey KJ , Mathew B , Bulman JC , *et al* Stem cell‐based treatments for Type 1 diabetes mellitus: bone marrow, embryonic, hepatic, pancreatic and induced pluripotent stem cells. Diabet Med. 2012; 29: 14–23. 21883442 10.1111/j.1464-5491.2011.03433.x

[48] Blyszczuk P , Asbrand C , Rozzo A , *et al* Embryonic stem cells differentiate into insulin‐producing cells without selection of nestin‐expressing cells. Int J Dev Biol. 2004; 48: 1095–104. 15602695 10.1387/ijdb.041904pb

[49] Lumelsky N , Blondel O , Laeng P , *et al* Differentiation of embryonic stem cells to insulin‐secreting structures similar to pancreatic islets. Science. 2001; 292: 1389–94. 11326082 10.1126/science.1058866

[50] Weissman IL . Translating stem and progenitor cell biology to the clinic: barriers and opportunities. Science. 2000; 287: 1442–6. 10688785 10.1126/science.287.5457.1442

[51] Jun HS , Yoon JW . Approaches for the cure of type 1 diabetes by cellular and gene therapy. Curr Gene Ther. 2005; 5: 249–62. 15853732 10.2174/1566523053544209

[52] Kim S , Shin JS , Kim HJ , *et al* Streptozotocin‐induced diabetes can be reversed by hepatic oval cell activation through hepatic transdifferentiation and pancreatic islet regeneration. Lab Invest. 2007; 87: 702–12. 17483848 10.1038/labinvest.3700561

[53] Nagaishi K , Ataka K , Echizen E , *et al* Mesenchymal stem cell therapy ameliorates diabetic hepatocyte damage in mice by inhibiting infiltration of bone marrow‐derived cells. Hepatology. 2014; 59: 1816–29. 24375439 10.1002/hep.26975

[54] Xia JL , Dai C , Michalopoulos GK , *et al* Hepatocyte growth factor attenuates liver fibrosis induced by bile duct ligation. Am J Pathol. 2006; 168: 1500–12. 16651617 10.2353/ajpath.2006.050747 PMC1606599

[55] Takahashi K , Yamanaka S . Induction of pluripotent stem cells from mouse embryonic and adult fibroblast cultures by defined factors. Cell. 2006; 126: 663–76. 16904174 10.1016/j.cell.2006.07.024

[56] Gennero L , Roos MA , Sperber K , *et al* Pluripotent plasticity of stem cells and liver repopulation. Cell Biochem Funct. 2010; 28: 178–89. 20232487 10.1002/cbf.1630

[57] Calafiore R , Basta G . Stem cells for the cell and molecular therapy of type 1 diabetes mellitus (T1D): the gap between dream and reality. Am J Stem Cells. 2015; 4: 22–31. 25973328 PMC4396156

[58] Soria B , Roche E , Berna G , *et al* Insulin‐secreting cells derived from embryonic stem cells normalize glycemia in streptozotocin‐induced diabetic mice. Diabetes. 2000; 49: 157–62. 10868930 10.2337/diabetes.49.2.157

[59] Wang HW , Lin LM , He HY , *et al* Human umbilical cord mesenchymal stem cells derived from Wharton's jelly differentiate into insulin‐producing cells *in vitro* . Chin Med J. 2011; 124: 1534–9. 21740812

[60] Phadnis SM , Joglekar MV , Dalvi MP , *et al* Human bone marrow‐derived mesenchymal cells differentiate and mature into endocrine pancreatic lineage *in vivo* . Cytotherapy. 2011; 13: 279–93. 21039304 10.3109/14653249.2010.523108

[61] Zhao Y , Mazzone T . Human cord blood stem cells and the journey to a cure for type 1 diabetes. Autoimmun Rev. 2010; 10: 103–7. 20728583 10.1016/j.autrev.2010.08.011

[62] Park KS , Kim YS , Kim JH , *et al* Trophic molecules derived from human mesenchymal stem cells enhance survival, function, and angiogenesis of isolated islets after transplantation. Transplantation. 2010; 89: 509–17. 20125064 10.1097/TP.0b013e3181c7dc99

[63] Abdi R , Fiorina P , Adra CN , *et al* Immunomodulation by mesenchymal stem cells: a potential therapeutic strategy for type 1 diabetes. Diabetes. 2008; 57: 1759–67. 18586907 10.2337/db08-0180 PMC2453631

[64] Chen LB , Jiang XB , Yang L . Differentiation of rat marrow mesenchymal stem cells into pancreatic islet beta‐cells. World J Gastroenterol. 2004; 10: 3016–20. 15378785 10.3748/wjg.v10.i20.3016 PMC4576264

[65] Oh SH , Muzzonigro TM , Bae SH , *et al* Adult bone marrow‐derived cells trans‐differentiating into insulin‐producing cells for the treatment of type I diabetes. Lab Invest. 2004; 84: 607–17. 15034596 10.1038/labinvest.3700074

[66] Timper K , Seboek D , Eberhardt M , *et al* Human adipose tissue‐derived mesenchymal stem cells differentiate into insulin, somatostatin, and glucagon expressing cells. Biochem Biophys Res Commun. 2006; 341: 1135–40. 16460677 10.1016/j.bbrc.2006.01.072

[67] Pessina A , Eletti B , Croera C , *et al* Pancreas developing markers expressed on human mononucleated umbilical cord blood cells. Biochem Biophys Res Commun. 2004; 323: 315–22. 15351739 10.1016/j.bbrc.2004.08.088

[68] Ende N , Chen R , Reddi AS . Transplantation of human umbilical cord blood cells improves glycemia and glomerular hypertrophy in type 2 diabetic mice. Biochem Biophys Res Commun. 2004; 321: 168–71. 15358230 10.1016/j.bbrc.2004.06.121

[69] Ende N , Chen R , Reddi AS . Effect of human umbilical cord blood cells on glycemia and insulitis in type 1 diabetic mice. Biochem Biophys Res Commun. 2004; 325: 665–9. 15541340 10.1016/j.bbrc.2004.10.091

[70] Lee RH , Seo MJ , Reger RL , *et al* Multipotent stromal cells from human marrow home to and promote repair of pancreatic islets and renal glomeruli in diabetic NOD/scid mice. Proc Natl Acad Sci USA. 2006; 103: 17438–43. 17088535 10.1073/pnas.0608249103 PMC1634835

[71] Urban VS , Kiss J , Kovacs J , *et al* Mesenchymal stem cells cooperate with bone marrow cells in therapy of diabetes. Stem Cells. 2008; 26: 244–53. 17932424 10.1634/stemcells.2007-0267

[72] Zhang D , Jiang W , Liu M , *et al* Highly efficient differentiation of human ES cells and iPS cells into mature pancreatic insulin‐producing cells. Cell Res. 2009; 19: 429–38. 19255591 10.1038/cr.2009.28

[73] Hua H , Shang L , Martinez H , *et al* iPSC‐derived beta cells model diabetes due to glucokinase deficiency. J Clin Investig. 2013; 123: 3146–53. 23778137 10.1172/JCI67638 PMC3696557

[74] Thatava T , Kudva YC , Edukulla R , *et al* Intrapatient variations in type 1 diabetes‐specific iPS cell differentiation into insulin‐producing cells. Mol Ther. 2013; 21: 228–39. 23183535 10.1038/mt.2012.245 PMC3538320

[75] Tateishi K , He J , Taranova O , *et al* Generation of insulin‐secreting islet‐like clusters from human skin fibroblasts. J Biol Chem. 2008; 283: 31601–7. 18782754 10.1074/jbc.M806597200

[76] Hori Y , Gu X , Xie X , *et al* Differentiation of insulin‐producing cells from human neural progenitor cells. PLoS Med. 2005; 2: e103. 15839736 10.1371/journal.pmed.0020103 PMC1087208

[77] Chang CM , Kao CL , Chang YL , *et al* Placenta‐derived multipotent stem cells induced to differentiate into insulin‐positive cells. Biochem Biophys Res Commun. 2007; 357: 414–20. 17433254 10.1016/j.bbrc.2007.03.157

[78] Soria B , Gauthier BR , Martin F , *et al* Using stem cells to produce insulin. Expert Opin Biol Ther. 2015; 15: 1469–89. 26156425 10.1517/14712598.2015.1066330

[79] Karaoz E , Okcu A , Unal ZS , *et al* Adipose tissue‐derived mesenchymal stromal cells efficiently differentiate into insulin‐producing cells in pancreatic islet microenvironment both *in vitro* and *in vivo* . Cytotherapy. 2013; 15: 557–70. 23388582 10.1016/j.jcyt.2013.01.005

[80] Barcelos LS , Duplaa C , Krankel N , *et al* Human CD133^+^ progenitor cells promote the healing of diabetic ischemic ulcers by paracrine stimulation of angiogenesis and activation of Wnt signaling. Circ Res. 2009; 104: 1095–102. 19342601 10.1161/CIRCRESAHA.108.192138 PMC2821014

[81] Prochazka V , Gumulec J , Chmelova J , *et al* Autologous bone marrow stem cell transplantation in patients with end‐stage chronical critical limb ischemia and diabetic foot. Vnitr Lek. 2009; 55: 173–8. 19378841

[82] Vojtassak J , Danisovic L , Kubes M , *et al* Autologous biograft and mesenchymal stem cells in treatment of the diabetic foot. Neuro Endocrinol Lett. 2006; 27 (Suppl. 2): 134–7. 17159798

[83] Cho KA , Ju SY , Cho SJ , *et al* Mesenchymal stem cells showed the highest potential for the regeneration of injured liver tissue compared with other subpopulations of the bone marrow. Cell Biol Int. 2009; 33: 772–7. 19427913 10.1016/j.cellbi.2009.04.023

[84] Yan Y , Xu W , Qian H , *et al* Mesenchymal stem cells from human umbilical cords ameliorate mouse hepatic injury *in vivo* . Liver Int. 2009; 29: 356–65. 19141029 10.1111/j.1478-3231.2008.01855.x

[85] Ali G , Mohsin S , Khan M , *et al* Nitric oxide augments mesenchymal stem cell ability to repair liver fibrosis. J Transl Med. 2012; 10: 75. 22533821 10.1186/1479-5876-10-75 PMC3419634

[86] Maeda M , Takami T , Terai S , *et al* Autologous bone marrow cell infusions suppress tumor initiation in hepatocarcinogenic mice with liver cirrhosis. J Gastroenterol Hepatol. 2012; 27 (Suppl. 2): 104–11. 22320927 10.1111/j.1440-1746.2011.07016.x

[87] Bell GI , Broughton HC , Levac KD , *et al* Transplanted human bone marrow progenitor subtypes stimulate endogenous islet regeneration and revascularization. Stem Cells Dev. 2012; 21: 97–109. 21417581 10.1089/scd.2010.0583

[88] Anzalone R , Lo Iacono M , Loria T , *et al* Wharton's jelly mesenchymal stem cells as candidates for beta cells regeneration: extending the differentiative and immunomodulatory benefits of adult mesenchymal stem cells for the treatment of type 1 diabetes. Stem Cell Rev. 2011; 7: 342–63. 20972649 10.1007/s12015-010-9196-4

[89] Ezquer M , Ezquer F , Ricca M , *et al* Intravenous administration of multipotent stromal cells prevents the onset of non‐alcoholic steatohepatitis in obese mice with metabolic syndrome. J Hepatol. 2011; 55: 1112–20. 21356258 10.1016/j.jhep.2011.02.020

[90] Si Y , Zhao Y , Hao H , *et al* Infusion of mesenchymal stem cells ameliorates hyperglycemia in type 2 diabetic rats: identification of a novel role in improving insulin sensitivity. Diabetes. 2012; 61: 1616–25. 22618776 10.2337/db11-1141 PMC3357293

[91] Lee KD , Kuo TK , Whang‐Peng J , *et al* *In vitro* hepatic differentiation of human mesenchymal stem cells. Hepatology. 2004; 40: 1275–84. 15562440 10.1002/hep.20469

[92] Kuroda Y , Kitada M , Wakao S , *et al* Bone marrow mesenchymal cells: how do they contribute to tissue repair and are they really stem cells? Arch Immunol Ther Exp. 2011; 59: 369–78. 10.1007/s00005-011-0139-9 21789625

[93] Li M , Guo K , Vanella L , *et al* Stem cell transplantation upregulates Sirt1 and antioxidant expression, ameliorating fatty liver in type 2 diabetic mice. Int J Biol Sci. 2015; 11: 472–81. 25798066 10.7150/ijbs.10809 PMC4366645

[94] Wang Y , Huso DL , Harrington J , *et al* Outgrowth of a transformed cell population derived from normal human BM mesenchymal stem cell culture. Cytotherapy. 2005; 7: 509–19. 16306013 10.1080/14653240500363216

[95] Miura M , Miura Y , Padilla‐Nash HM , *et al* Accumulated chromosomal instability in murine bone marrow mesenchymal stem cells leads to malignant transformation. Stem Cells. 2006; 24: 1095–103. 16282438 10.1634/stemcells.2005-0403

[96] Lu YR , Yuan Y , Wang XJ , *et al* The growth inhibitory effect of mesenchymal stem cells on tumor cells *in vitro* and *in vivo* . Cancer Biol Ther. 2008; 7: 245–51. 18059192 10.4161/cbt.7.2.5296

[97] McCall MD , Toso C , Baetge EE , *et al* Are stem cells a cure for diabetes? Clin Sci (Lond). 2010; 118: 87–97. 19807695 10.1042/CS20090072

[98] Tian LL , Yue W , Zhu F , *et al* Human mesenchymal stem cells play a dual role on tumor cell growth *in vitro* and *in vivo* . J Cell Physiol. 2011; 226: 1860–7. 21442622 10.1002/jcp.22511

[99] Gluckman E . History of cord blood transplantation. Bone Marrow Transplant. 2009; 44: 621–6. 19802032 10.1038/bmt.2009.280

[100] Vija L , Farge D , Gautier JF , *et al* Mesenchymal stem cells: stem cell therapy perspectives for type 1 diabetes. Diabetes Metab. 2009; 35: 85–93. 19230736 10.1016/j.diabet.2008.10.003

[101] Yang L , Li S , Hatch H , *et al* *In Vitro* Transdifferentiation of adult hepatic stem cells into pancreatic endocrine hormone‐producing cells. Proc National Acad Sci USA. 2002; 99: 8078–83. 10.1073/pnas.122210699 PMC123023 12048252

